# Optimized Langendorff perfusion system for cardiomyocyte isolation in adult mouse heart

**DOI:** 10.1111/jcmm.15773

**Published:** 2020-11-04

**Authors:** Haotong Li, Chungeng Liu, Minghui Bao, Weijing Liu, Yu Nie, Hong Lian, Shengshou Hu

**Affiliations:** ^1^ State Key Laboratory of Cardiovascular Disease National Center for Cardiovascular Disease Chinese Academy of Medical Sciences and Peking Union Medical College Fuwai Hospital Beijing China; ^2^ Department of Cardiovascular Surgery Tongji Medical College Union Hospital Huazhong University of Science and Technology Wuhan China; ^3^ Department of Cardiology Peking University First Hospital Beijing China; ^4^ Department of Biochemistry and Molecular Biology Shanxi Medical University Taiyuan China

**Keywords:** adult mouse heart, cardiomyocyte isolation, Langendorff perfusion system

## Abstract

With the rapid development of single‐cell sequencing technology, the Langendorff perfusion system has emerged as a common approach to decompose cardiac tissue and obtain living cardiomyocytes to study cardiovascular disease with the mechanism of cardiomyocyte biology. However, the traditional Langendorff perfusion system is difficult to master, and further, the viability and purity of cardiomyocytes are frequently unable to meet sequencing requirements due to complicated devices and manipulate processes. Here, we provide an optimized Langendorff perfusion system with a simplified and standardized operating protocol which utilizes gravity as the perfusion pressure, includes a novel method for bubbles removing and standardizes the criteria for termination of digestion. We obtained stable cardiomyocyte with high viability and purity after multiple natural gravity sedimentation. The combination of the optimized Langendorff perfusion system and the multiple natural gravity sedimentation provides a stable system for isolating adult mouse heart, which will provide higher‐quality cardiomyocytes for further experiments.

## 1 INTRODUCTION

Cardiovascular disease is the leading cause of death worldwide, with myocardial infarction or heart failure accounting for the majority.^1^ For basic researches on myocardial infarction or heart failure, in addition to functional experiments using animal models, there are a large number of in vitro mechanism studies that require the approaches to obtain myocardial cells for primary cell culture or sequencing.^2^, ^3^ With the rapid development of single‐cell technology, researches in the cardiovascular field have also converted from bulk sequencing to single‐cell sequencing.^4^ To obtain a single‐cell suspension of the whole heart or a certain type of cardiac cells, as a solid organ, the heart needs to be digested and the cells are required to be purified. At present, the common methods of isolating adult mouse heart include the classic Langendorff method and the non‐classic Langendorff‐free method.^5^, ^6^, ^7^ The main principle of the two methods is to use the anatomical characteristics of the ‘aorta—coronary sinus ostium—coronary artery’ of the mouse heart to perfuse digestion buffer for enzymatic hydrolysis. The main difference between the two approaches is that the Langendorff method is retrograde perfusion, while the Langendorff‐free method is antegrade perfusion.

Although in recent years, much research has been carried out to optimize and innovate experimental techniques for isolating adult mouse heart,^2^, ^6^, ^7^, ^8^, ^9^ there still exist many problems. The Langendorff apparatus is too complicated, the cell isolating operation is difficult to be learned, and consequently, the viability and purity of the cells frequently fail to meet the sequencing requirements, and the repeatability is poor with large batch differences.^4^ Mastering the Langendorff perfusion system requires months of continuous practice, which is not conducive to the popularization and application of the technology in the cardiovascular field. Standardizing, optimizing and simplifying the experimental process will shorten the learning cycle, facilitate the application and promote the repeatability.

In this study, we provide a detailed protocol of an optimized Langendorff method with a modified and simplified Langendorff apparatus using gravity as perfusion pressure for isolating adult mouse heart, which makes it easy to learn and operate and improves the viability and purity of the target cell population isolated from adult mouse heart.

## 2 MATERIALS AND METHODS

### 2.1 Mice

All animal experiments were conducted following the Guide for the Use and Care of Laboratory Animals. All animal protocols were approved by the Institutional Animal Care and Use Committee (IACUC), Fuwai Hospital, Chinese Academy of Medical Sciences. The C57BL/6J WT adult (8 ~ 12 weeks) mice with similar bodyweight were obtained from Vital River Laboratory Animal Technology Co. Ltd. for experiments.

### 2.2 Flow cytometry

For flow cytometry analysis of the purity of isolated cardiomyocytes, cells after calcium reintroduction were resuspended in 1× phosphate buffer saline (PBS) with 5% bovine serum albumin (BSA) and 4% paraformaldehyde and 0.3% Triton‐X 100 for fixation/permeabilization. Cell suspension was then centrifuged at 100 *g* for 3 minutes and resuspended in 1 × PBS and incubated with the anti‐α‐actinin (Sarcomeric)—PE antibody (Miltenyi Biotec; 1:100) for 30 minutes keep in a dark place at 4°C with gentle rotation and then washed three times with 1 × PBS. Samples were analysed by flow cytometry (BD FACS Arial II, BD bioscience), and the following cell quantitation and data processing were performed using FlowJo V.10 (Tree Star, Inc.).

### 2.3 Cardiomyocyte nuclei staining

Isolated cardiomyocytes were incubated with Hoechst 33342 Staining Dye Solution (Abcam, 1:1000) in a cell incubator at 37°C for 30 minutes. Discard the supernatant and wash cardiomyocytes three times with 1 × PBS. The fluorescence microscope (Zeiss, Axio Observer A1) was used to observe stained cardiomyocyte nuclei at a wavelength of 405 nm.

### 2.4 Statistical analysis

All data are expressed as the mean ± standard deviation (SD). Student's *t* test was used for comparison between two groups, whereas one‐way ANOVA was used for comparisons among more than two groups. *P* < 0.05 was considered statistically significant.

### 2.5 Solutions and buffers

Details can be seen in Table S1.

## 3 RESULTS AND DISCUSSION

### 3.1 Modified and simplified Langendorff apparatus makes successful perfusion easier

**Figure 1:**
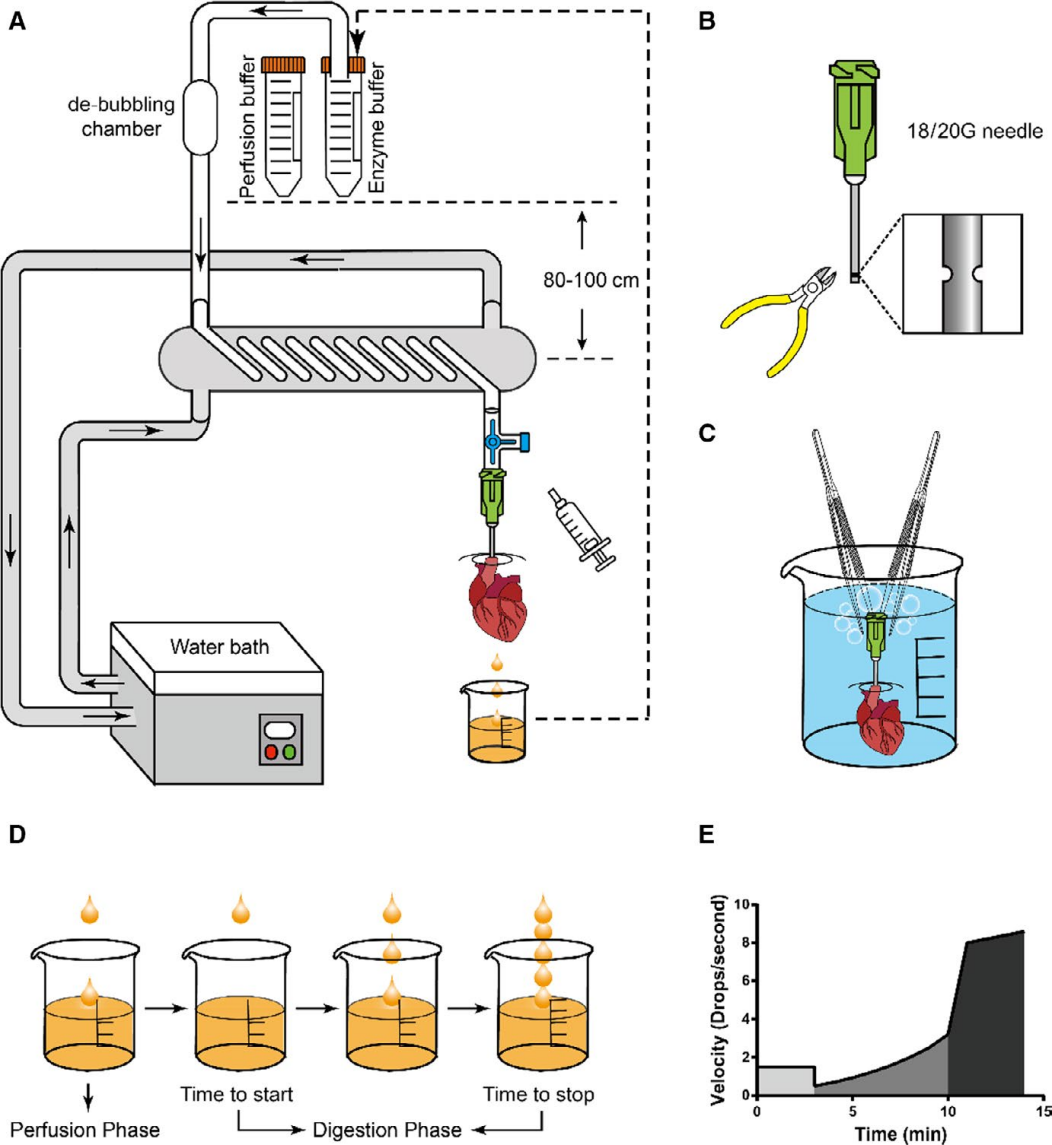
Modified and simplified Langendorff apparatus and method. A, Schematic diagram of the modified and simplified Langendorff apparatus. B, 18/20G flat‐headed needle with a circular groove at about 2 mm from the tip by using the sharp‐nosed pliers. C, Effective de‐bubbling method. D‐E, Determination of perfusion status and standardization of the criteria for termination of digestion

The traditional Langendorff method is complicated and cumbersome. Most of the problems come from the equipment and protocol itself rather than the proficiency of the operator.^7^ If there is no simple and operable experimental equipment or operation procedure, the success of the experiment of isolating cardiomyocytes from adult heart mainly depends on the operator's skills and proficiency. To simplify this technology and promote its stability, we optimized the Langendorff equipment (Figure 1A), improved the needle used for aortic intubation (Figure 1B) and ameliorated the de‐bubbling method (Figure 1C). Technical differences in adult mouse heart isolation and purification among laboratories and this study have been summarized in Table 1.

**Table 1:** Technical differences in adult mouse heart isolation and purification among laboratories

Reference	Timothy D. O'Connell et al, Methods Mol Biol, 2007^8^	Justin Judd et al, JOVE‐J VIS EXP, 2016^6^	Matthew Ackers‐Johnson et al, Circ Res, 2016^7^	Gregory A. Quaife‐Ryan et al, Circulation, 2017^2^	Bilin Liu et al, JMCC, 2019^9^	This study
Mouse strain	NA	ICR (CD1)	C57BL/6J	ICR (CD1)	C57BL/6N	C57BL/6J
Perfusion method	Retrograde Langendorff perfusion	Retrograde Langendorff perfusion	Langendorff‐free	Retrograde Langendorff perfusion	Retrograde Langendorff perfusion	Modified Langendorff perfusion
Perfusion pressure	Perfusion pump	Perfusion pump	By hand, press syringe	NA	Peristaltic pump	Gravity
Perfusion buffer PH	7.0	NA	7.8	NA	7.2 ~ 7.8	7.4
Digestion time	About 11 min	10 ~ 15 min	12 ~ 25 min	8 min	30 min	10 ~ 12 min
Criteria for termination of digestion	Heart should appear pale and flaccid	Heart begin slouching	Noticeable reduction in resistance to injection pressure, loss of shape and rigidity	Until the hearts appeared waxy in colour and flaccid to the touch	NA	Depend on the velocity of the liquid
Mechanical dissociation	Cut into small pieces with fine forceps and blow with plastic transfer pipets 3 ~ 5 min	Cut into small pieces with fine forceps and put them in a petri dish in a 37°C incubator, agitate every 2 min	Using sharp scissors and 1 mL pipette, 2 min	Minced with fine scissors into small pieces and triturated to release cells	Cut into small pieces and gentle agitation, 5 min	Cut into small pieces with fine forceps and blow with plastic transfer pipets no more than 2 min
Purification	Centrifuge for 3 min at 20 *g*	Gravity sedimentation; settle by gravity for 15 min	Sequential gravity settling for 10 min in 4 mL fresh Perfusion buffer per 15 mL tube	Centrifugation, 30 *g* × 3 min	Centrifugation, 20 *g* × 3 min	Gravity sedimentation; settle by gravity for 10 min; repeat 4 times
Yield per heart	1 ~ 1.5 × 10^6^ CMs	0.5 ~ 1 × 10^6^ CMs	≤1 × 10^6^ CMs	NA	0.6 × 10^6^ CMs	0.9 ~ 1.2 × 10^6^ CMs
Rod‐shaped rate	67%	NA	81% ± 6%	NA	70%	83% ± 3%
Purity	NA	NA	NA	NA	NA	97%
Advantages	Detailed steps and a lot of practical experience sharing	The ability to culture the isolated cardiomyocytes long term	Simple equipment	Adult mouse post‐MI 3 d also can be isolated by the classical method	Compared the method between isolate rat and mouse heart	Simplified and modified equipment and easy operation, high quality and purity

Abbreviations: CM, cardiomyocyte; MI, myocardial infarction; NA, not available.

First, we have to remove the heart from the mouse chest cavity by cutting the aortic arch as close as possible to the innominate artery, and after that, we need to suspend the aorta to the needle and fasten it with silk thread as soon as possible. This step is crucial to the success of the experiment. In this step, we need to ensure the two points are completed. One point is that the depth of the aortic cannula should be appropriate, and it should not be lower than the aortic valve or enter the left ventricle (Figure S1). Another point is to avoid air bubbles entering the aorta as much as possible. To ensure the success of intubation, we used an 18G flat‐headed needle that is similar to the aortic diameter of adult mice (8 ~ 12 weeks), and sharp‐nosed pliers were used to clamp the needle at about 2 mm from the tip to form a circular groove (Figure 1B). The circular groove can facilitate the intubation depth control and prevent the heart from slipping out during intubation. We connected the syringe with the perfusion buffer to the optimized needle and fixed the syringe with an iron stand; the trimmed heart was placed in a petri dish with perfusion buffer at 4°C under a stereomicroscope. Before intubation, there are two points that we need to make sure: (a) the heart is completely immersed in perfusion buffer in the petri dish; (b) to push the syringe to eliminate the possible air bubbles in the needle. The aortic cannulation process should be completed within 2 minutes if possible.

After the intubation is completed, the needle needs to be connected to the Langendorff device through the tri‐branch tube (Figure 1). During this operation, once air bubbles enter the needle or aorta, they will cause air embolism of the coronary artery and lead to the death of cardiomyocytes. Therefore, we optimized the method to remove bubbles (Figure 1C). We filled a 50 mL beaker with perfusion buffer and put the heart with the connected needle into the beaker. If there are bubbles in the aorta or needle, they will float in an upright position. We use two microforceps to guide and remove the air bubbles (usually visible to the naked eye) in the aorta or the needle. The successful sign of de‐bubbling is that there are no air bubbles visible at the head of the needle. Additionally, when the heart with the needle are placed in the liquid, they will sink to the bottom of the beaker. After evacuating, the air bubbles in the needle and the aorta open the tri‐branch tube and make the perfusion buffer flow down by using the negative pressure suction effect of the syringe. When there is a continuous and stable stream out of the tri‐branch tube, connect the needle with the heart to the tri‐branch tube and start perfusion. After the perfusion buffer was infused for 3 minutes, move the tube to a 50 mL centrifuge tube containing enzyme buffer. Our effective de‐bubbling method is the key to avoid air embolism, which is one of the most common reasons for experiment failure.

Conventional methods mostly use peristaltic pumps as the power source of the perfusion buffer.^6^, ^8^ However, our optimized device uses gravity as perfusion pressure, which is more in line with the biological characteristics of heart.^10^ The heart has a higher tension in the undigested state, while the fully digested heart has a lower tension due to the hydrolysis of the extracellular matrix. When gravity is used as the pressure, the perfusion pressure on the heart will not change with the progress of the digestion process. In contrary to gravity perfusion, the peristaltic pump method maintains a constant flow rate and, thus, may cause excessive pressure in the initial stage of digestion and cause damage to cardiomyocytes.^11^ We have compared the classical method using peristaltic pumps to provide perfusion pressure with our optimized Langendorff system to isolate adult mouse cardiomyocytes, and the results revealed that the rate of rod‐shaped cardiomyocytes in the optimized system (83.10% ± 3.27%, means ± SD) is significantly higher than in the classical system (70.97% ± 3.98%, means ± SD) (Figure S2). In our Langendorff perfusion system, due to the height between the centrifuge tube with buffers and the hanging heart directly determines the coronary perfusion pressure, we calculated the recommended height for mice and rat according to their coronary perfusion pressure (Table S2).^11^, ^12^ Another advantage of the gravity method is that it can easily determine whether the perfusion is successful in the early stage of digestion. Because the enzyme buffer contains a small amount of Ca^2+^, but not in the perfusion buffer, so when the enzyme buffer just enters the coronary artery, it will cause a sudden increase in myocardial tension. Consequently, the phenomenon which will be observed by us is that the speed of the drops dripping from the bottom of the heart suddenly slows down (Figure 1D‐E). The appearance of this phenomenon indicates successful aortic cannulation and perfusion.

### 3.2 Standardization of the criteria for termination of digestion

The digestion degree of the heart greatly affects the results of subsequent experiments.^13^ Either insufficient or excessive digestion will lead to unreliable experimental results. For example, counting the number and evaluating the ploidy of cardiomyocytes in the field of cardiac regeneration need strict criterion of digestion to keep the consistency and accuracy of experiments.^14^ Currently, the digestion time of heart is mainly determined by the experience of operators. However, the fibrosis degree and ECM composition differ between animals of different species, different ages, different physiological or pathological conditions (such as physiological adult mice, physiological elderly mice, pathological mice with surgical model, or mice with certain gene knockout or overexpressing),^15^ and thus, the optimal digestion time must be different from each other. Therefore, it is particularly important to develop a simple and effective approach to judge the degree of digestion.

In our optimized Langendorff perfusion system, we can easily determine when to stop digestion by observing the speed change of the droplets flowing from the bottom of the hanging heart (Figure 1D‐E). In the initial stage of perfusion, the flow rate is maintained at about 2 drops per second (5 mL/min) without change. Once the enzyme buffer enters the heart, the flow rate will suddenly slow down (about 2 mL/min; can be easily recognized by the naked eye). After a few minutes, as the cardiac extracellular matrix hydrolyses, the heart tension decreases, and the droplet flow rate gradually increases. Finally, when the heart is fully digested, the flow rate will suddenly increase which may be due to the rupture of the coronary system or atrium (no less than 25 mL/min). Generally, digestion can be terminated for another 2 minutes at this time. We observed that shorter digestion time (about 3 minutes shorter than standard time) led to lower cardiomyocyte viability (56.47% ± 3.13%, means ± SD). No significant difference of cardiomyocyte viability was found between the longer time group (3 minutes longer than the standard time, 77.30% ± 5.43%, means ± SD) and the standard time group (83.47% ± 3.17%, means ± SD). Although proper extension of digestion time may not affect cardiomyocyte viability, inevitable excessive digestion may occur and affect the physiological state of cardiomyocytes.^16^ This simple method may well standardize the time for termination of digestion and can be generalized to various conditions of mice.

### 3.3 Multiple natural gravity sedimentation improves cardiomyocyte purity and viability

**Figure 2:**
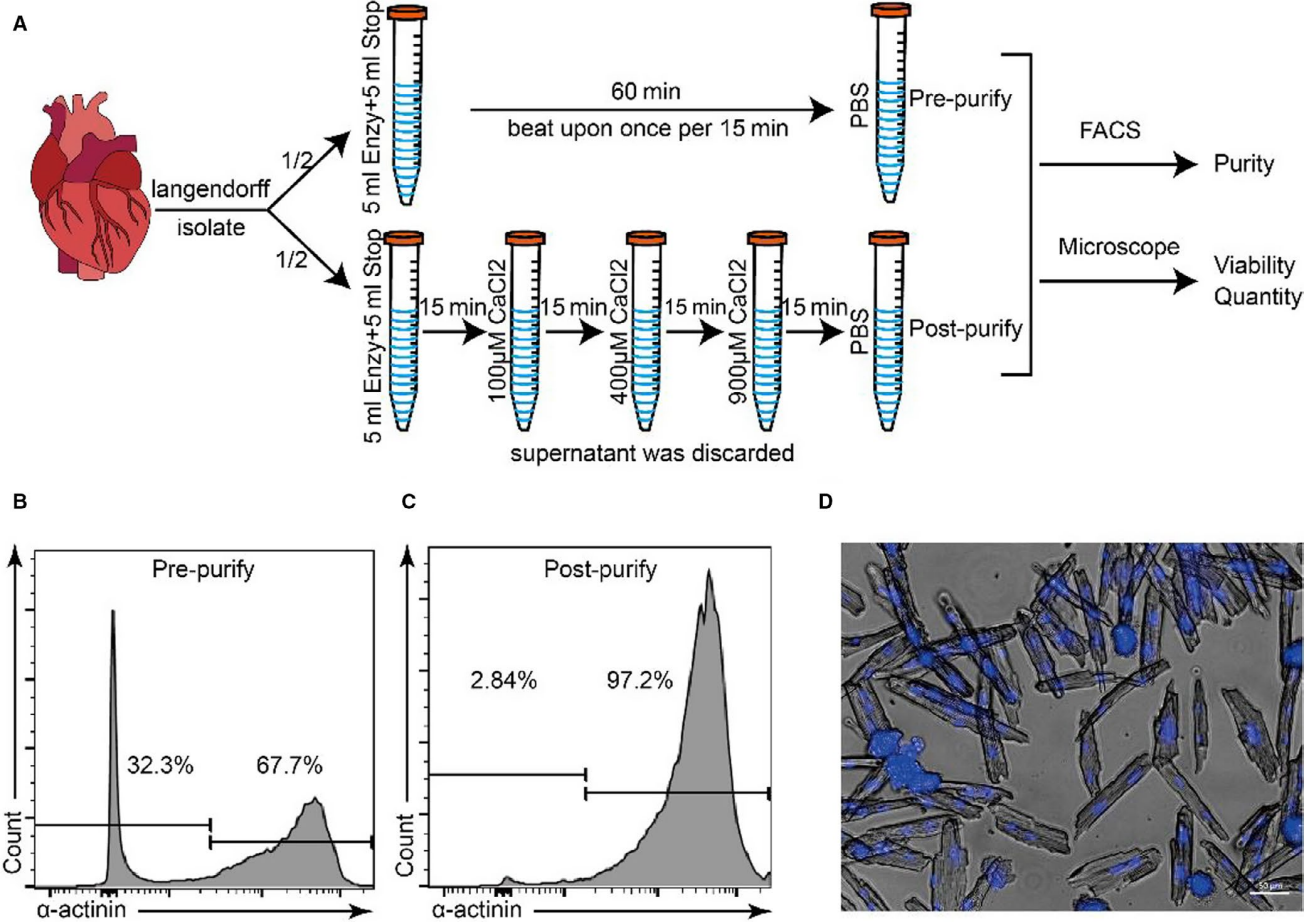
Multiple natural gravity sedimentation approach improves cardiomyocyte purity and viability. A, Schematic of multiple natural gravity sedimentation approach. B‐C, Detection of cardiomyocyte purity by flow cytometry pre‐purify (63.83% ± 4.06%, means ± SD) and post‐purify (96.83% ± 0.40%, means ± SD) (n = 3). D, Freshly isolated adult mouse cardiomyocytes with rod‐shaped morphology, sharp‐edged membranes and intact nuclei stained with Hoechst 33342. The scale bar is 50 μm

To reducing data variability in further experiments, we need to obtain cardiomyocytes with higher viability and purity.^4^ Instead of using low‐speed centrifugation, we purified cardiomyocytes by multiple natural gravity sedimentation, which bring cardiomyocytes of greater viability and purity. We shred the digested heart into small pieces and then pipette several times until we cannot see the massive myocardial tissue. Next, we perform natural sedimentation by gravity and reintroduce of calcium of gradient concentration to cardiomyocytes at the same time to bring them to a normal physiological state. We prepared three 15 mL centrifuge tubes, added 10 mL stop buffer to each centrifuge tube, and added CaCl_2_ solution, adjusted to 100, 400 and 900 μm Ca^2+^ concentration, respectively (Figure 2A). The stop buffer with gradient Ca^2+^ liquid needs to be changed every 15 minutes. Because cardiomyocytes are much larger than other cardiac cells, gravity will lead the cardiomyocytes to sink to the bottom faster, and meanwhile, the highly active cardiomyocytes gathered up due to the aggregation effect. After that, flow cytometry can be used to test the purity of cardiomyocytes before sequencing. The proportion of cardiomyocytes measured before purification was only 63.8% ± 4.1% (means ± SD), while reached 96.8% ± 0.4% (means ± SD) after purification (Figure 2B,C). Staining cardiomyocyte nuclei with Hoechst 33342 and observing under a microscope, we can see those rod‐shaped cardiomyocytes with sharp‐edged membranes and intact nuclei (Figure 2D). These results suggested that the purity and viability of cardiomyocytes have been well optimized in terms of viability (83.10% ± 3.27%, means ± SD), purity (96.83% ± 0.40%, means ± SD) and cell count (0.9 ~ 1.2 × 10^6^), which may reach the single‐cell sequencing requirements. The Percoll gradient separation and centrifugation can be used for further purification of cardiomyocytes and further increasing the proportion of viable cells.^17^

## 4 CONCLUSION

We have optimized the Langendorff perfusion system and provided a stable and feasible method for isolating adult mouse heart by using gravity as perfusion pressure, establishing an effective de‐bubbling method and standardizing the criteria for termination of digestion. The cardiomyocytes with high purity and viability achieving from our optimized approach would facilitate the acquirement of high‐quality cardiomyocytes for sequencing or other follow‐up experiments and contribute to more stable experimental results of cardiomyocytes isolation.

## 5 LIMITATION

This method is only conducted on mice under physiological conditions and has not been applied in the isolation and purification of mouse cardiac cells under pathological conditions (such as myocardial infarction or transverse aortic constriction surgical models).

## CONFLICT OF INTEREST

The authors declare that they have no conflicts of interest with the contents of this article.

## AUTHOR CONTRIBUTIONS

**Haotong Li:** Data curation (lead); Methodology (lead); Visualization (lead); Writing‐original draft (equal). **chungeng Liu:** Data curation (supporting); Methodology (equal); Validation (equal). **Minghui Bao:** Writing‐original draft (equal). **Weijing LIU:** Methodology (supporting); Writing‐review & editing (supporting). **Yu Nie:** Conceptualization (equal); Funding acquisition (supporting). **Hong Lian:** Conceptualization (equal); Funding acquisition (lead); Writing‐review & editing (equal). **Shengshou Hu:** Project administration (lead); Supervision (lead); Writing‐review & editing (equal).

## Supporting information

Supplementary Material

## Data Availability

The data that support the findings of this study are available from the corresponding author upon reasonable request.
