# Supplementary material for: Optimized Langendorff perfusion system for cardiomyocyte isolation in adult mouse heart
Source: J Cell Mol Med. 2020 Nov 4;24(24):14619–25. doi: 10.1111/jcmm.15773 (PMC7754046; doi:10.1111/jcmm.15773)
Supplement: Supplementary file 1 — Supplementary Material [file JCMM-24-14619-s001.docx]

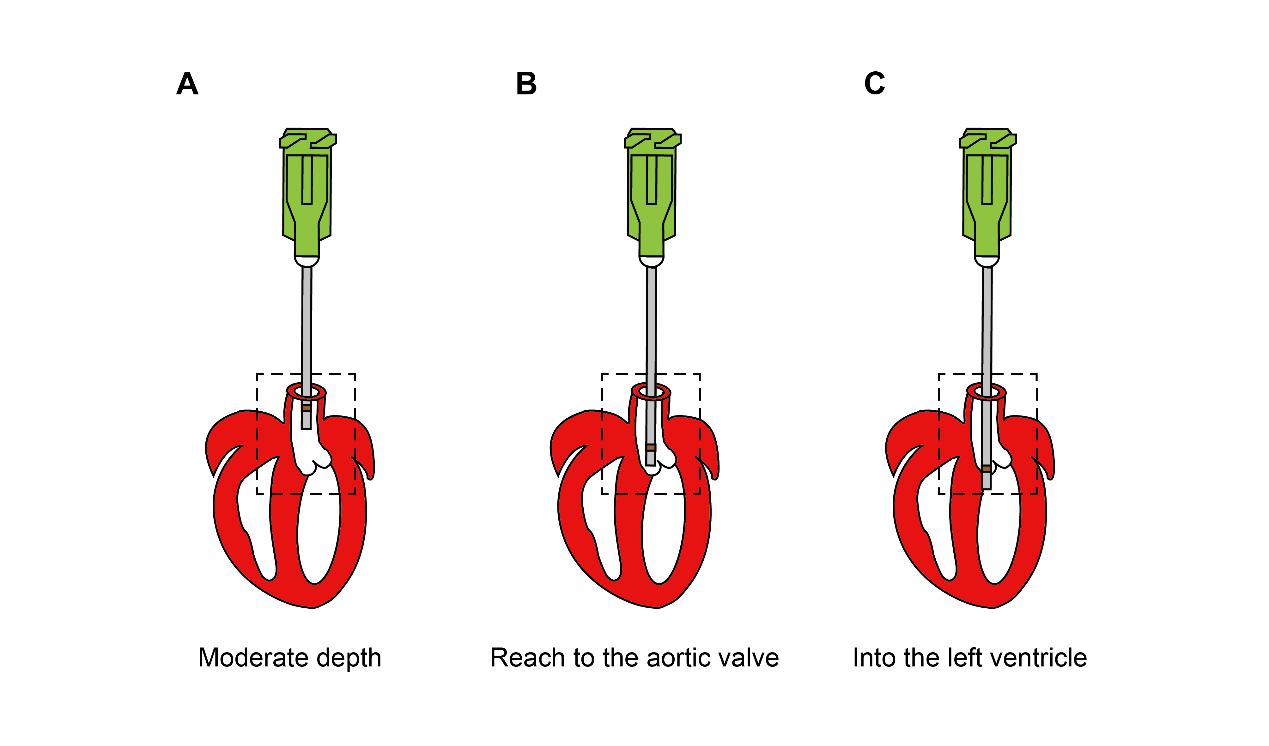


**Supplementary Figure 1. Schematic of moderate depth of aortic cannulation. A,** Moderate depth (about 2 mm) was perfect to replicate the method. **B,** The tip of the needle reaches to the aortic valve may impairs the effect of perfusion. **C,** The tip of the needle inserted into the left ventricle result in perfusion failure.

­­­­­­
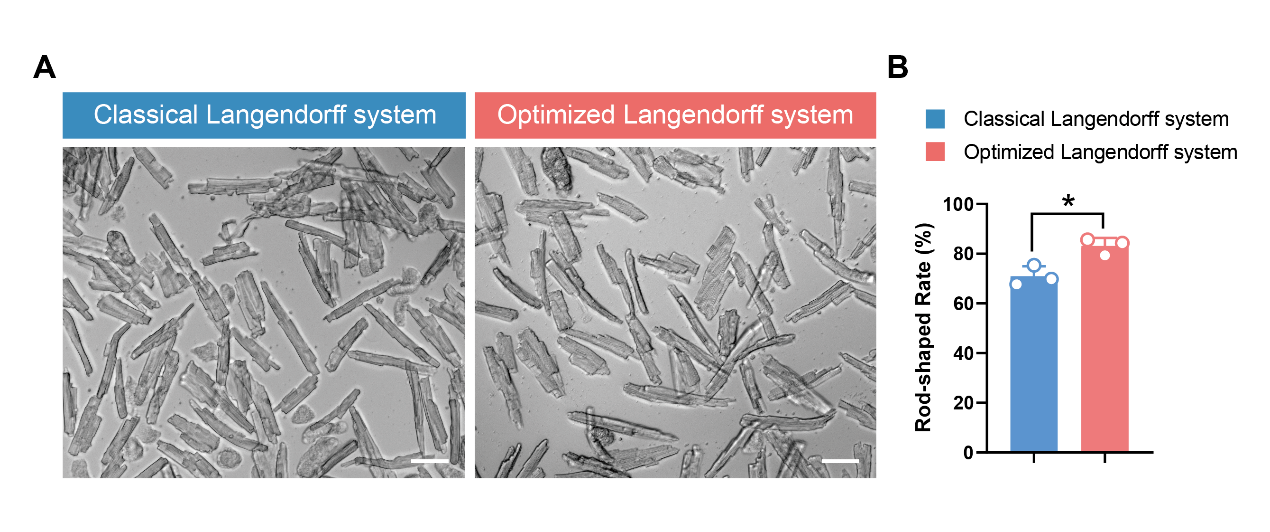
 **Supplementary Figure 2. Optimized Langendorff system improves cardiomyocyte viability.** **A,** Adult mouse cardiomyocytes stained with Hoechst 33342 isolated by classical Langendorff system using peristaltic pumps (left; scale bar, 50 μm) and optimized Langendorff system (right; scale bar, 50μm). **B,** Quantification of viability of cardiomyocytes isolated by the classical and optimized Langendorff system (n=3, per group). Statistical significance was determined by Student’s t-test and data is presented as mean ± SD. *P<0.05.


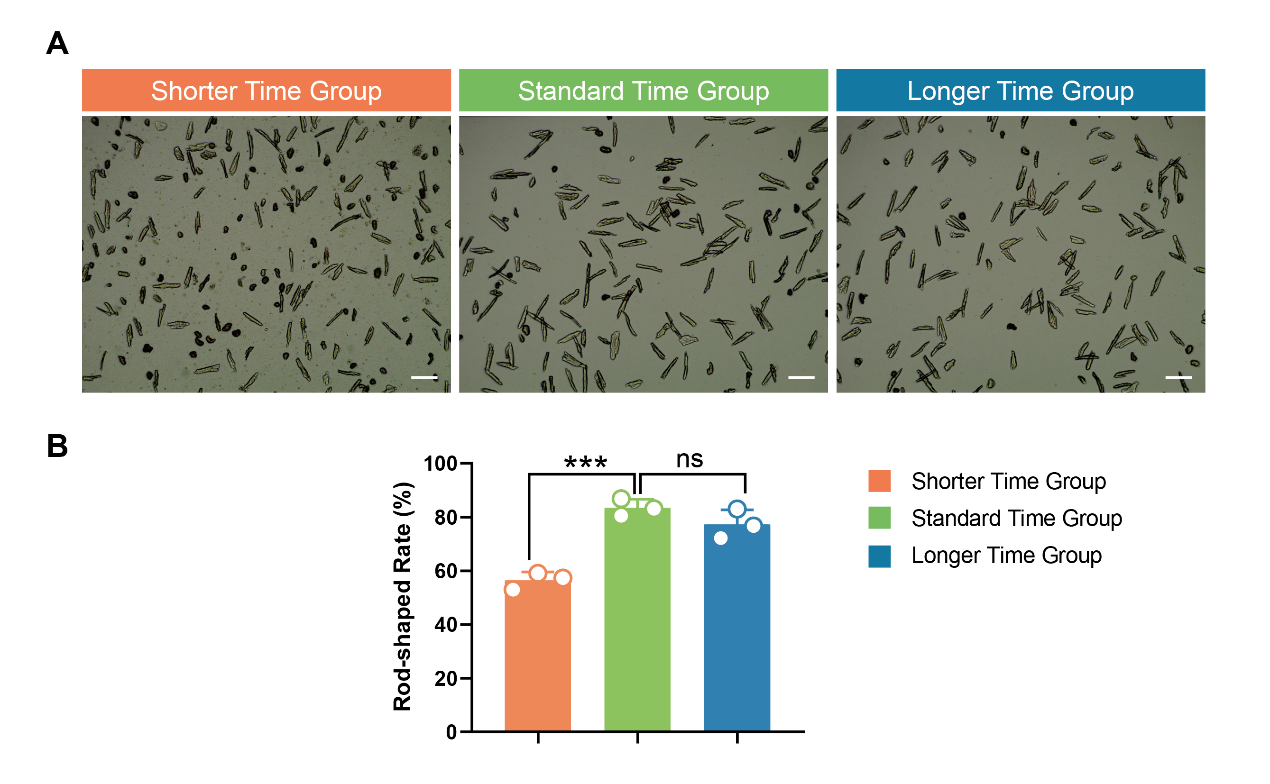


**Supplementary Figure 3. Standardized digestion time is necessary for isolating adult mouse cardiomyocytes.** **A,** Brightfield images show adult mouse cardiomyocytes post-isolation with different digestion time. The left is the shorter time group (about 3 min shorter than the standard time); the middle is the standard time group (the standard time is determined by our method); the right is the longer time group (3 min longer than the standard time). Scale bar, 100 μm. **B,** Quantification of viability of isolated cardiomyocytes with different digestion time (n=3, per group). Statistical significance was determined by one-way ANOVA and data is presented as mean ± SD. ***P<0.001; ns, not statistically significant.

**Supplementary Table 1.** Solution and buffers

| **Perfusion buffer (1L)** | | | | |
| --- | --- | --- | --- | --- |
| Component | Amount (g/L) | Final concentration (μM) | Company | Catalogue number |
| NaCl | 7.013 | 120 | *Sigma-Aldrich* | S6191 |
| KCl | 0.403 | 5.4 | *Sigma-Aldrich* | P9541 |
| MgCl_2_ | 0.114 | 1.2 | *Sigma-Aldrich* | M8266 |
| NaH_2_PO_4_ | 0.040 | 0.3 | *Sigma-Aldrich* | S8282 |
| Glucose | 1.802 | 10 | *Sigma-Aldrich* | G8270 |
| HEPES | 4.766 | 20 | *Sigma-Aldrich* | 54457 |
| BDM | 1.011 | 10 | *Sigma-Aldrich* | B0753 |
| Taurine | 1.252 | 10 | *Sigma-Aldrich* | 86329 |
| Adjust the pH with 2 M NaOH to 7.40 | | | | |
|  | | | | |
| **Enzyme buffer (50ml/per adult mouse heart)** | | | | |
| Component | Amount | Final concentration | Company | Catalogue number |
| Perfusion buffer | 50 ml | — | — | — |
| Collagenase II | 50 mg | 1 mg/ml | *Worthington* | LS004176 |
| Protease XIV | 3 mg | 0.06 mg/ml | *Sigma-Aldrich* | P5147 |
| CaCl_2_ (100 M) | 15 μl | 30 μM | *Sigma-Aldrich* | C7902 |
|  | | | | |
| **Stop buffer (50ml/per adult mouse heart)** | | | | |
| Component | Amount | Final concentration | Company | Catalogue number |
| Perfusion buffer | 50 ml | — | — | — |
| BSA | 250 mg | 0.5% | *Sigma-Aldrich* | A7906 |
| CaCl_2_ (100 M) | 25 μl | 50 μM | *Sigma-Aldrich* | C7902 |

**Supplementary Table 2.** Aortic pressure in different conditions and recommended height of centrifuge tube

|  | Mouse | Rat |
| --- | --- | --- |
| Coronary perfusion pressure (mmHg) | 60~80 | 70-100 |
| Recommended height between the centrifuge tube and the hanging heart (cm) | 80-100 | 95-135 |
| Calculation formula | Height (cm)≈(coronary perfusion pressure*133)/ (ρ*g*10)=1.35*coronary perfusion pressure | |

ρ, ρ_H2O_= 1.0 g/cm^3^; g= 9.8 m/s^2^
